# Understanding Challenges to Well-Being among Latina FarmWorkers in Rural Idaho Using in an Interdisciplinary, Mixed-Methods Approach

**DOI:** 10.3390/ijerph18010169

**Published:** 2020-12-29

**Authors:** Cynthia L. Curl, Lisa Meierotto, Rebecca L. Som Castellano

**Affiliations:** 1 Department of Community and Environmental Health, College of Health Sciences, Boise State University, Boise, ID 83725, USA; 2 School of Public Service, Boise State University, Boise, ID 83725, USA; lisameierotto@boisestate.edu; 3 Department of Sociology, College of Arts and Sciences, Boise State University, Boise, ID 83725, USA; rsomcastellano@boisestate.edu

**Keywords:** Latina, farmworkers, well-being, pesticides, biomonitoring

## Abstract

The aim of this study was to identify social, cultural and workplace-related risk factors affecting well-being among Latina farmworkers in rural Idaho. We recruited 70 Latina farmworkers from southwestern Idaho in 2019. We employed an inter-disciplinary, mixed-methods approach—including surveys, focus groups, interviews, and pesticide biomonitoring—to characterize multiple domains that influence well-being, including food security and access, housing conditions, social supports, access to medical care, and workplace safety. Six major themes emerged as primary challenges to Latina farmworkers’ well-being. In the public sphere, study participants identified these challenges as long working hours, concerns regarding pesticide exposure, and lack of enforcement of regulatory protections. Participants’ concerns regarding pesticide exposure were underscored by biological sampling results; multiple biomarkers of pesticide exposure were detected in all samples, with the highest concentrations measured in samples collected from women who reported mixing, loading or applying pesticides. Within the private sphere, food security and provisioning, childcare responsibilities, and social isolation were identified as significant challenges to well-being. Gender, ethnicity, and geography emerged as important, intersecting statuses that shaped the life experiences of these agricultural workers. Our findings suggest that gender may play a particularly critical role in the unique challenges facing Latina farmworkers. As a result, the services and regulations needed to support well-being in this population may be highly specific, and almost certainly include attention to work–family dynamics, pesticide exposure, and social connections.

## 1. Introduction

A range of factors shape the well-being of Latinx farmworkers in the United States (US). Their work is often contingent and low-paid [1,2,3]. They labor in, and live in, rural places with fewer resources [4]. They have limited access to insurance and physical and mental health care [5], and face well-documented occupational health and safety risks [2,6]. Family separation, social hierarchies, insufficient social networks and other forms of social suffering also influence Latinx farmworker well-being [1,7,8,9]. Further, immigration politics have been central to shaping farmworker livelihoods in the US, and immigration status affects access to federally-funded safety nets which can improve health and welfare [1,10].

Collectively, these factors create an environment in which Latinx farmworkers are likely to suffer—physically, socially and psychologically. However, another form of marginalization often goes unrecognized in consideration of the experience of Latinx farmworkers: gender. In recent decades, there has been a marked increase in the proportion of women in the US agricultural workforce [11], growing from 25% in 1989 to 32% in 2016 [12,13]. However, there is a limited body of research that examines the experiences of women farmworkers, particularly Latina farmworkers [14]. These workers deserve attention not only because their numbers are increasing across the agricultural industry, but also because the additional marginalization associated with gender may lead to further disadvantage.

Women farmworkers are often more economically vulnerable, relative to men, in part because of gender-segregated work [15]. Further, women farmworkers remain primarily responsible for childcare and household labor in the private sphere [14]. Labor in the public and private spheres may conflict, decreasing well-being [16], and Latina farmworkers are more likely to report work–family conflict and less support from supervisors [15]. Women farmworkers are also more likely to experience elevated depressive symptoms, relative to men [17], and the prevalence of depression is high in general among Latina women in farmworker families [18,19,20].

Latina farmworkers also may experience increased barriers to occupational health and safety, such as ill-fitting protective equipment or reduced access to safety training [21]. A study of 220 Latina women in farmworker families, a third of whom themselves worked in agriculture, found that just 50 participants (38%) reported always having access to proper safety equipment [22]. These barriers to occupational health and safety may result in increased exposure to pesticides; in the US, two studies employing the Sentinel Event Notification System for Occupational Risks (SENSOR)-Pesticides program found that acute pesticide poisonings were nearly twice as prevalent in women working in agriculture compared to men [23,24]. Other studies have found higher levels of pesticide exposure among Latina nursery workers compared to controls [25], and Latina farmworkers were found to have urinary pesticide metabolite levels above those measured in nationally representative samples, although these levels were not higher than Latina non-farmworkers in the same study [26]. 

While Latina farmworkers may, therefore, face multiple, varied challenges to health and well-being, there remains more to be learned about their perspectives on these challenges. In this manuscript, we employ the concept of structural vulnerability to frame our understanding of the well-being of Latina farmworkers. Structural vulnerability asserts that existing social hierarchies shape the degree to which an individual is vulnerable within a community (or society), and calls attention to the political and social contexts within which people labor [27]. The factors noted above, which may influence well-being among Latina farmworkers, are an outcome of structural vulnerability, and stem from inequalities based on gender, race and ethnicity, socioeconomic status, and space and place.

The connection between structural inequality and farmworker well-being has been made elsewhere, such as in the work of Holmes and Horton, who note the ways in which economic and social forces shape physical, social and psychological well-being among Latinx farmworkers [28,29]. However, less attention been given to the ways in which gender shapes well-being among Latina farmworkers. Yet as noted above, gender inequalities may be a key driver in understanding the vulnerabilities Latina farmworkers face, including those related to well-being [30].

In the research presented here, we aimed to characterize factors contributing to and challenging well-being among a cohort of Latina farmworkers in Idaho. We developed a mixed-method approach to gather information on multiple dimensions of well-being, and further aimed to understand aspects of well-being that matter most to Latina farmworkers. 

## 2. Materials and Methods

This study aimed to identify challenges to well-being, and to assess related social, cultural and workplace-related risk factors, among Latina farmworkers in Idaho. While we recognize the complexities of well-being, many scholars have argued for its usefulness as a tool for measuring quality of life [31,32,33]. In assessing well-being and related risk factors, we employed an interdisciplinary approach including four components: surveys, focus groups, interviews, and biological monitoring. All aspects of this study were reviewed and approved by our university’s Institutional Review Board (IRB).

### 2.1. Recruitment, Enrollment and Consent

Our study sample included women aged 18 years or older who identified as Latina or Hispanic farmworkers during the screening process. Data collection occurred in either English or Spanish based on participant preference. All participants were recruited from southwest Idaho between October 2018 and June 2019. In-person recruitment occurred at local community-based organizations including Migrant and Farmworker Head Start programs, community events such as local festivals and health fairs, and churches and grocery stores. We committed in advance to sharing the findings of our study with community-based organizations, which we ultimately did in the form of a research report and through in-person presentations. Conducting research with a rural, vulnerable population has many challenges. In order to successfully recruit participants in this study, we worked in partnership with multiple trusted community organizations; included bilingual research staff in all study components; scheduled interviews and focus groups primarily on evenings and weekends; welcomed children during interviews, focus groups and survey completion; and, in some cases, provided childcare during study activities.

Consent was obtained separately for each form of data collection (surveys, focus groups, interviews, and biological monitoring, each discussed below). While all participants completed the survey component of the study, participants were allowed to opt in or out of the additional components. Grocery store gift cards ranging in value from $10 to $25 compensated participation in each component. The survey coversheet stated “If you agree to participate in this survey, please turn the page to begin,” and survey completion thus indicated consent to participate in this aspect of the research. No identifiers were collected as part of the survey. Following survey completion, women were asked whether they were willing to be contacted for participation in additional study components. Those who provided informed consent for additional components also provided their name and contact information (which was then linked to their survey IDs) to schedule future study procedures. All identifying information was coded and stored in a locked-file cabinet per IRB protocol. 

### 2.2. Surveys

The survey included six domains of inquiry: sociodemographics; food security and food access; housing conditions; social isolation; access to medical care; and occupational hazards. Survey items were based on several previously validated instruments, including the National Agricultural Workplace Survey [34], surveys of housing conditions and social isolation among farmworkers in the southeastern US [35,36], as well as a survey previously developed to assess food quality and availability among Latina farmworkers in Idaho [14]. This survey was in paper format (English and Spanish), was completed individually by the respondent, and required between 15 and 35 min to complete. 

### 2.3. Focus Groups

Study participants who indicated a willingness to be contacted for participation in focus groups were called within a week of survey completion. Focus group participants were also recruited via snowball sampling and flyers, which were posted in grocery stores and housing complexes proximate to farmworker community housing and shared among community partners. Five focus groups were held at community centers and restaurants in southwestern Idaho between January and May 2019. Focus group participants who had not previously completed the study survey were asked to do so during the focus group itself.

Focus groups were primarily conducted in Spanish and lasted between 60 and 120 min. A Spanish-speaking member of the research team attended each focus group to facilitate discussion with non-English speakers. Researchers also took detailed notes and/or audio recorded the sessions, and recordings were transcribed and translated to English. Discussion occurred through guided facilitation around participants’ own definitions of well-being and the dimensions of well-being of greatest concern and importance. Facilitated discussion also centered around how farm work influences well-being, and the strategies that farmworkers use to improve well-being. Participants were also asked to identify the relative importance of multiple aspects of well-being and how well-being varies seasonally in both facilitated discussion, and through activities. We provided meals for participants and any family members in attendance.

### 2.4. Interviews

Study participants willing to take part in the interview component of the study were contacted by phone to schedule the interviews. These semi-structured interviews occurred between March and June of 2019, lasted between 45 and 90 min, and occurred at a location of the participants’ choosing. Interview participants were asked a range of questions about their experiences with farm work, including benefits and challenges. Interviews were conducted in English or Spanish, based on participant preference. All interviews were audio-recorded, transcribed and translated as needed.

### 2.5. Biological Monitoring

Study participants who indicated a willingness to participate in the biological monitoring component of the study were called within a week of survey completion; others were approached directly during focus groups. Each participant was asked to provide two urine samples to reflect exposures both during the time of year when pesticides are not actively applied in Idaho agriculture (the “non-spray season”, defined as January 1 through April 14) and the active agricultural season during which pesticides are commonly sprayed (the “spray season”, defined as April 15 through June 30). Spray season designation was based on local farming practices in 2019. 

Samples were transported to our laboratory on ice, analyzed for specific gravity, and stored at −80 °C until overnight shipment on dry ice to an external laboratory. There, they were analyzed for eleven metabolites of common insecticides and herbicides [37]. These included the metabolites of four organophosphate insecticides, five pyrethroid insecticides, and two herbicides. Included with the sample shipment were six duplicate samples, which were each analyzed for all eleven biomarkers; laboratory analyses were blinded to the identity of these quality assurance (QA) samples. 

### 2.6. Advocate Interviews

We interviewed five farmworker advocates, identified as professionals who work in support of farmworkers and their families in southwestern Idaho. During these interviews, we asked about organizational mission, job description, and perceptions of the rewards and challenges among Latina farmworkers. The results of these interviews provided additional perspectives and context on the experiences of Latina farmworkers. 

### 2.7. Data Analysis

Survey data were entered into Microsoft Excel and duplicate data checks were made by two researchers to ensure the accuracy of the data entry. Survey data were analyzed using STATA 13 (StataCorp; College Station, TX, USA). Frequencies of missingness, counts of categorical responses, and summary statistics were generated for all items.

Focus group and interview data were analyzed using NVivo (QSR International, V12), a computer-assisted qualitative data analysis program. The qualitative data were coded using both inductive and deductive coding techniques. Multiple members of the research team worked together to develop a coding scheme. They then conducted line-by-line coding of the transcripts to assign codes to the text. They did so independently, and results were compared to ensure inter-coder reliability.

For biomonitoring results, we first calculated the relative percent difference (RPD) between duplicate pairs of QA samples and assessed the frequency of detection for all biomarkers. We decided a priori to focus our analysis on biomarkers with an RPD <40% and a detection frequency >50% based on the treatment of censored data recommended by Antweiler and Taylor [38]. For biomarkers detected in at least 50% of samples, concentrations below the detection limit were replaced with a value equal to the limit of detection divided by the square root of 2 [39]. Prior to all analyses, we employed specific gravity measurements to adjust for urinary dilution according to the method described by Chiu et al. [40]. We calculated summary statistics for all biomarkers and compared measured concentrations across seasons and across job tasks using the nonparametric Mann–Whitney U test for non-normally distributed data. We also identified outliers in the urinary biomonitoring dataset, defined as concentrations greater than or equal to 1.5 times the interquartile range (IQR) above the third quartile or 1.5 times the IQR below the first quartile. Extreme outliers were defined as three times the IQR above and below the third and first quartiles, respectively.

## 3. Results

### 3.1. Participant Characteristics

We recruited 70 women who identified as Latina farmworkers to participate in this study, all of whom completed the survey component. We conducted five focus groups with a subset of 22 of these women, and 11 women participated in the semi-structured interviews. We also collected 44 urine samples from 29 of the study participants. Table 1 shows the characteristics of the full cohort, as well as the characteristics of subsets completing each study component.

The average age of the study participants was 37.3 years old. On average, there were 2.7 children in each household, with a range of 0–6. Overall, 91% of the participants identified as Mexican, Mexican-American, or Chicana. Approximately half of the participants reported earning less than $19,999 per year. Participants indicated that their agricultural employment was not limited to the summer; a third of the participants reported engaging in farm work in winter months. Onions (75%) and corn (57%) were the most commonly worked crops. On average, respondents had worked in agriculture for 11 years. We observed little migration among our sample, given that 84% of the respondents reported living at their current home for the past 12 months, and 83% reported that they or their spouses had not traveled outside of the state for work in the past 12 months.

### 3.2. Emergent Themes Related to Well-Being and Work in the Public Domain

Findings from our focus groups and interviews indicated that women had complex feelings around agricultural work. On one hand, women expressed joy with working outside and with other women. One woman explained, “Es trabajo pesado. Pero bonito.” (“It is difficult work, but beautiful [work].”) One participant had worked in agriculture for several decades, since she was seven years old. She expressed contentedness with working with her family, explaining, “It was wonderful to work all together.” However, there were also many challenges that our participants expressed with their roles in the agricultural workforce, and our analyses indicated three primary themes related to these challenges: long working hours, concerns about pesticide exposure and lack of enforcement of regulatory protections. A representative quote regarding each of these challenges is provided in Table 2.

#### 3.2.1. Long Hours

During the interviews and focus groups, farm work was often described as “hard” and as involving long days. Participants described how their long working hours made it difficult to access medical care and engage in household labor. As one participant emphasized, “no hay tiempo para la familia” (there is no time [outside of work] for family). These descriptions during the interviews and focus groups were consistent with survey results; 7 of 52 women (13.5%) who answered survey questions regarding barriers to medical care reported that getting time off from work was an important barrier to receiving medical care. One farmworker advocate stated that lack of time is a significant challenge for Latina farmworkers, saying “They don’t have time for themselves…they definitely have no time for their children…that’s when you start seeing like a domino effect of like bad eating habits, you know or like they’re not being a participant in the children’s activity. And I think our moms are afraid to be selfish.”

#### 3.2.2. Pesticide Exposure

Study participants were also concerned about pesticide exposure at work. They described not knowing whether the fields in which they worked had been sprayed with pesticides, and said that they had to work wherever they were told, without any information about pesticide applications.

The results of our biological monitoring show that there may be reason for concern about pesticide exposures in this population. Six of the pesticide biomarkers we measured met our criteria for quality assurance and frequency of detection: malathion dicarboxylic acid (MDA), para-nitrophenol (PNP), 3,5,6-tricholor-2-pyridinol (TCPY), 3-phenoxybenzoic acid (3-PBA), the trans isomer of 3-(2,2-dichlorovinyl)-2-2-dimethylcyclopropane carboxylic acid (trans-DCCA), and 2,4-dichlorophenoxyacetic acid (2,4-D). Five of these, MDA, PNP, TCPY, 3-PBA and trans-DCCA, represent exposure to organophosphate and pyrethroid insecticides, which have been associated with neurological dysfunction in agricultural workers [30]. The metabolite 2,4-D represents exposure to an herbicide that has been associated with cancer in agricultural workers [31]. Every urine sample collected contained detectable concentrations of MDA, PNP, 3-PBA, and 2,4-D; 91% of the samples contained detectable concentrations of TCPY and trans-DCCA (see Table 3). 

While not statistically significant, an exploratory analysis of outlying values within the biomonitoring data indicated some potentially important trends. Specifically, we observed that the samples containing the highest concentrations of five of these six biomarkers were collected during the agricultural spray season. Further, the most extreme outlying concentrations of MDA, 3-PBA, trans-DCCA and 2,4-D were measured in samples collected during the spray season from women who reported that they loaded, mixed or applied pesticides.

#### 3.2.3. Regulatory Protections

Under the Worker Protection Standard (WPS), agricultural employers are required to provide all workers with supplies for routine and emergency decontamination in the event of pesticide exposures, including “plenty of” soap and single use towels, and either one gallon of water per worker or 3 gallons of water per handler at the beginning of each work period [41]. Accessible toilets are also required [41]. However, according to the survey results, 7% of our participants did not have access to toilets every day, and 21% reported that employers did not provide water to wash hands every day. This lack of basic sanitation also emerged as a theme in the focus groups and interviews.

Employers are also required to give workers proper notification about the timing of pesticide applications and spraying and to provide workers with training in pesticide safety, including advanced training requirements for those workers who mix, load or apply pesticides [41]. As noted above, women commonly reported that they were not given any notification of when pesticide sprays had occurred in the fields where they worked. Five women in our cohort reported performing pesticide-handling activities, but just two of these women reported that they had received the required advanced pesticide handler training.

We measured the highest concentrations of MDA, 3-PBA, trans-DCCA, and 2,4-D in samples collected from women who reported handling pesticides. Further, the highest concentration of MDA (which was also the highest concentration among all pesticides and metabolites measured), was measured in a sample collected from a handler who reported that she did not receive pesticide handler training.

### 3.3. Emergent Themes Related to Well-Being and Work in the Private Domain

Our analyses also indicated three primary themes related to the well-being of farmworkers as they navigate the private sphere. These themes were concerns about food security and provisioning, childcare responsibilities and social isolation. A representative quote regarding each of these challenges is provided in Table 4.

#### 3.3.1. Food Security and Food Provisioning

When completing the survey, 87% of the women in our study answered “no” when asked if they were hungry but could not eat due to finances (in the past year). On its surface, this might indicate food security. However, based on survey results about their experiences over the past year, 61% reported that it was “often” or “sometimes” true that food did not last and they did not have money for more food, and 68% reported that it was “often” or “sometimes” true that balanced meals were unaffordable. While respondents predominantly reported paying for food with cash (93%), use of social supports was evident, with 22% using the Supplemental Nutrition Assistance Program (SNAP), 38% using Women, Infants and Children (WIC) services, and 94% of women with school aged children taking advantage of the free and reduced-price meals made available through the National School Lunch Program (NSLP). 

Farmworker advocates we spoke with also noted food-related challenges. One advocate working in childcare explained, “We get a lot of children here at our center that come in the morning hungry and it takes a while for us to notice who the children are that are always hungry in the morning and we try to make sure they eat and we feed them good before they leave. Because like I said, some of them don’t get fed until mom and dad come home or mom comes home. So, they go to the babysitters and they’re not eating there until they get picked up by mom and dad. So, sometimes it’s hard especially for a single mother trying to put food on the table if they have more than one child. It’s hard and it’s getting expensive.”

#### 3.3.2. Childcare and Domestic Responsibilities

Challenges around labor in the private sphere emerged as a critical theme in the data. Significant parts of the focus group discussions centered on the challenges of working in agriculture while raising children. Focus group participants also discussed gender inequality in the distribution of household labor. They reported that the unequal sharing of household tasks, including childcare, becomes particularly challenging for women during planting and harvesting, which requires long work hours.

Interview findings reflect some of these same challenges. Several participants identified strain with managing household responsibilities and their paid employment; this was even more pronounced for single mothers. One woman explained, “women who are single mothers…say that it’s difficult for them to pay rent or groceries because sometimes they don’t have families either, they are alone.” This statement underscores the connectedness of challenges to well-being faced by these women, as it integrates concerns over childcare, finances, and social isolation.

#### 3.3.3. Social Isolation

Social isolation emerged as an important theme among our study participants. According to the survey data, 67% of survey respondents reported spending time with family on every or most days, but when asked if they had friends or relatives to count on, including for financial assistance, 36% said “no,” and an additional 13% reported that they “did not know.” In addition, 35% reported that they experienced belonging “not very strongly or not at all” to their community and an additional 25% reported “do not know.” The region of Idaho from which we recruited study participants is heavily agricultural and rural, and somewhat isolated, and may present limited opportunity for community connections.

## 4. Discussion

This study aimed to describe challenges to well-being among a growing, yet understudied, population of Latina farmworkers. While significant literature exists describing the health-related risks faced by men who labor in agriculture [2,5,6,7], far less attention has been paid to the experiences of women in this same workforce. Building from our conceptual framework, our findings suggest that gender may play a critical role in the unique challenges facing farmworkers, as several of the themes that emerged in our work had very specific gendered aspects.

For instance, Latina farmworkers in this study frequently described the challenges of balancing the demands of agricultural work with their household responsibilities. In particular, childcare responsibilities—both the act of caring for children and the work involved in acquiring other forms of childcare during their work shifts—played a central role in the data. Women in this study also consistently expressed concerns around food security and food provisioning, for which they felt primary responsibility. These challenges and concerns may stem from gendered responsibilities which are an outcome of social hierarchies.

Consistent with these findings, previous research has found Latina farmworkers to experience greater stress and anxiety than Latinas who were employed in other occupations [30]. Further, employed Latinas (both farmworkers and non-farmworkers) were found to experience greater stress and anxiety than unemployed Latinas [30]. The authors of this work suggest several potential factors as possible causes of this increased stress and anxiety, including work–family balance and the “significant domestic responsibilities” these women shoulder.

Even concerns around pesticide exposure may have a gendered component, as women may be less likely to receive pesticide safety training than men and may be less likely to be issued personal protective equipment [21]. Female farmworkers may also be more susceptible to adverse health effects associated with pesticide exposures than their male counterparts [42], particularly during hormonal-based processes such as pregnancy, lactation and menopause [43]. In previous studies, pregnant farmworkers have expressed concern that pesticide exposure could be hazardous to pregnancy health [44]. While the sample size for the biological monitoring component of this study was relatively small, we did measure the highest exposures in samples collected during the agricultural spray season from women who reported mixing and applying pesticides. These outlying measurements were more than an order of magnitude higher than the 95th percentile of exposures measured in women and Mexican-Americans in the National Health and Nutrition Examination Survey [45,46,47], and notably higher for 3-PBA and *trans*-DCCA compared to the highest levels measured among migrant male farmworkers in Sonora, Mexico [48] (see Table 3). Further, the single highest exposure was measured in one such woman who reported that she never received any pesticide safety training.

Strengths of this study include a mixed-methods approach that allowed us to investigate various aspects of well-being using multiple measures, including surveys, interviews, focus groups, and pesticide biomonitoring. We also benefited from strong relationships with community-based organizations, which allowed us to recruit a reasonably sized cohort from a difficult to reach population. However, we acknowledge that this study was limited by the fact that we recruited a convenience sample, which is not necessarily representative of the Latina farmworker population in the US or even in the region of southwestern Idaho from which we recruited our participants. Furthermore, although we never intended for all study participants to take part in all of the study components, we recognize that each of the additional components was only completed by a subset of the overall cohort.

## 5. Conclusions

There is a need for additional research into the experiences of women farmworkers, and, in particular, Latina farmworkers. The findings of this study suggest that such research is crucial, both as the percent of our agricultural workforce comprised by women continues to grow and as we see evidence that the experiences of these women may differ from those of the men with whom they work. Our results suggest that the services and regulations needed to support the well-being of women farmworkers may be highly specific to this population, and almost certainly include attention to work–family dynamics, including childcare, and other gendered responsibilities, such as food provisioning. This is likely distinct from the more traditional needs attributed to farmworking populations—all of which still exist for these women—which only serves to underscore the multiple challenges these women face.

## Figures and Tables

**Table 1:** Participant characteristics in the study cohort of Latina Farmworkers recruited from Southwestern Idaho in 2019 [(N%) or Mean (SD)].

Characteristic	Total Cohort (*n* = 70) ^a^	Focus Group (*n* = 22) ^b^	Interview (*n* = 11)	Biomonitoring (*n* = 29)
Age (years) (mean, SD)	37.3 (10.8) ^c^	38.7 (13.7) ^c^	42.0 (13.8)	39.5 (10.6) ^c^
Race ^d^				
White	18 (26%)	4 (19%)	2 (18%)	7 (24%)
Non-White	27 (39%)	9 (43%)	6 (55%)	10 (35%)
Missing	24 (35%)	8 (38%)	3 (27%)	12 (41%)
Ethnicity ^d^				
Mexican-American	6 (9%)	4 (19%)	1 (9%)	3 (10%)
Mexican	56 (81%)	15 (71%)	9 (82%)	26 (90%)
Chicana	1 (1%)	1 (5%)	1 (9%)	1 (3%)
Other	7 (10%)	2 (10%)	0 (0%)	0 (0%)
Missing	1 (1%)	1 (5%)	0 (0%)	1 (3%)
Income (household, annually)				
Less than $19,999	34 (49%)	9 (43%)	4 (36%)	8 (28%)
$20,000–$49,999	26 (38%)	7 (33%)	6 (55%)	15 (52%)
$50,000 or more	1 (1%)	1 (5%)	0 (0%)	1 (3%)
Missing	8 (12%)	4 (19%)	1 (9%)	5 (17%)
Total number in household	5.1 (2.0)	5.1 (2.2)	5.2 (2.0)	5.3 (2.2)
Number of children living in household	2.7 (1.5)	2.3 (1.3)	2.7 (1.4)	2.6 (1.3)
Total years worked in agriculture (mean, SD)	11 (11)	11 (13)	15 (17)	11 (11)
Months worked in agriculture (in past year) (mean, SD)	7 (5)	8 (3)	7 (4)	7 (4)
Number (%) of women who report working in agriculture during each season ^d,e^				
Spring	46 (67%)	16 (76%)	7 (64%)	21 (72%)
Summer	53 (77%)	19 (90%)	9 (82%)	21 (72%)
Fall	35 (51%)	13 (62%)	5 (46%)	15 (52%)
Winter	23 (33%)	9 (43%)	5 (46%)	11 (38%)
Number (%) of women who report working with each of the following crops ^d,f^				
Onion	52 (75%)	20 (95%)	10 (91%)	21 (72%)
Corn	39 (57%)	17 (81%)	8 (73%)	17 (59%)
Potatoes	23 (33%)	6 (28%)	5 (46%)	9 (31%)
Hops	15 (22%)	4 (19%)	4 (36%)	7 (24%)
Grapes	16 (23%)	2 (10%)	3 (27%)	5 (17%)
Mint	18 (26%)	7 (33%)	2 (18%)	5 (17%)
Sugarbeets	10 (14%)	4 (19%)	3 (27%)	3 (10%)
Lived at current residence for past 12 months				
Yes	58 (84%)	17 (81%)	9 (82%)	22 (76%)
No	7 (10%)	2 (10%)	2 (18%)	4 (14%)
Missing	4 (6%)	2 (10%)	0 (0%)	3 (10%)
Traveled (or spouses traveled) outside of Idaho for farm work in past 12 months				
Yes	10 (14%)	3 (14%)	1 (9%)	4 (14%)
No	57 (83%)	17 (81%)	10 (91%)	23 (79%)
Missing	2 (3%)	1 (5%)	0 (0%)	2 (7%)

^a^ One survey excluded from analysis due to all missing data. ^b^ One focus group participant excluded from analysis due to all missing data. ^c^ One participant excluded from this calculation (improbable value, reported 2018 as year of birth). ^d^ Participants could select more than one answer. ^e^ Spring defined as March, April, May; Summer defined as June, July and August; Fall defined as September, October, November; Winter defined as December, January, February. ^f^ No participants reported working with soy or barley, fewer than 5 participants reported working with peas, hay, dairy, or beef.

**Table 2:** Representative quotes from focus groups and interviews with Latina farmworkers and farmworker advocates in southwestern Idaho in 2019. These quotes typify emergent themes related to work in the public sphere.

Emerging Theme	Representative Quote
Concerns about Long Work Hours	“When we go in at 5:30 or 6:00 [am], and we don’t get home until 6:00 [pm]. Because by the time I head home, it’s dinner, it’s—I have the girls, I have to pick them up…And so, it’s a very long day.” She further explained, “I think a lot of people don’t understand how hard it is to get up at 3:00 in the morning and get going and not go to bed until 11:00.”
Concerns about Pesticide Exposure	“Sometimes we would start vomiting and or faces would feel very itchy and we would get out but [the boss] would tell us, ‘Go back in because that is not hazardous.’ Things like that.”
Lack of Enforcement of Regulatory Protections	“And even when you have a hard time with the bathrooms because they don’t clean them very often. Many times you have to hold it because there are more than 100 people there using them all week. Just imagine. That’s one of the things that is very unpleasant but you have to put up with it because you don’t have any other choice.”

**Table 3:** Summary of pesticide biological monitoring (*n* = 29 women, 44 urine samples) results for Latina farmworkers recruited from southwestern Idaho in 2019.

Biomarker Metrics	Organophosphate Metabolites			Pyrethroid Metabolites		Herbicides
	MDA	PNP	TCPY	3-PBA	*trans*-DCCA	2,4-D
Frequency of Detection (%)	100%	100%	91%	100%	91%	100%
Mean (ng/mL)	2.3	0.9	0.6	1.2	1.4	1.2
95th Percentile (ng/mL)	4.3	2.2	1.9	3.2	3.8	1.6
Maximum Value (ng/mL)	51.7 ^a^	3.1	2.5	11.8 ^a^	23.4 ^a^	31.1 ^a^
NHANES 95th Percentile (Women) [31,32,33] (ng/mL)	2.1	6.9	8.4	4.4	3.0	8.8
NHANES 95th Percentile (Mexican-Americans) [31,32,33] (ng/mL)	1.7	17	5.8	1.2	1.4	26.4

^a^ Sample provided by a pesticide handler. Abbreviations: MDA: malathion dicarboxylic acid; PNP: para-nitrophenol; TCPY: 3,5,6-tricholor-2-pyridinol; 3-PBA: 3-phenoxybenzoic acid; *trans-*DCCA: the trans isomer of 3-(2,2-dichlorovinyl)-2-2-dimethylcyclopropane carboxylic acid; 2,4-D: 2,4-dichlorophenoxyacetic acid; and NHANES: National Health and Nutrition Examination Survey.

**Table 4:** Representative quotes from focus groups and interviews with Latina farmworkers and farmworker advocates in southwestern Idaho in 2019. These quotes typify emergent themes related to work in the private sphere.

Emerging Theme	Representative Quote(s)
Food security and food provisioning	“Yes, there was a time like that [when we ran out of food] three years ago during Christmas. I remember that I only had a ramen noodle soup. I told them [my kids], ‘You eat it.’ And they said, ‘No, mom, look, we can all share it.’ And we shared it between the three of us. Yes, there was a very difficult time.”
Childcare and Domestic Responsibilities	“The woman still comes home, and takes care of the man, and feeds them, and does the laundry. It is a big toll on a woman, I believe, to be an agricultural worker.”
Social Isolation	One woman explained that nine of her eleven siblings live in Mexico. When asked about family or community support, she stated, “Well, not help me if I need support, no. No, because I am very reserved person. If I need this or that, I don’t tell anyone.”

## Data Availability

The data presented in this study are available upon request from the corresponding author. The data are not publically available due to privacy restrictions and the personal and identifying nature of the transcripts of the interviews and focus groups.
